# Editorial - AfJEM Dec 2025

**DOI:** 10.1016/j.afjem.2025.100930

**Published:** 2025-11-24

As 2025 draws to a close, it gives me great pleasure to pen this editorial and celebrate some of the accomplishments of the African Journal of Emergency Medicine (AfJEM) as we head into our 15th year of the journal in 2026. It’s a new world of publishing since 2011, and AfJEM has weathered the journey from an era of printed issues to where we are now, a well-regarded, fully indexed, open-access journal with contents available to anyone with internet access. That’s no small accomplishment for the field and the journal, as we see many new journals struggling and falling by the wayside. The credit lies firstly with you, the authors across the continent who are the primary drivers of research, but also with the large reviewer team that peer reviews and holds each and every submission to a high standard. And lastly to the editorial team, entirely unfunded, who manage the process. In the last year we have embarked on expanding and revitalizing our team, and successfully recruited no less than 11 new Associate Editors from around the continent, which not only expands the spread of the journal to new institutions and domains, but also gives some of the long standing Associate Editors, many of whom have been with the journal from the early days the opportunity to step up to our International Advisory Board. We thank you all for your dedication and we believe this will bring a new era to the journal.

It’s been a busy year, with submissions to the journal up, as well as our accepted article numbers up – following this bumper issue, we will have published over 70 articles in 2025 which marks a return to the numbers we were seeing 4–5 years ago. What has changed in that time is that the authorship of articles has swung to a strong African focus and authorship, driven by journal policies and research expansion.

In this issue we see a fantastic spread of articles which talks to the broad and growing research domains in Emergency Care across Africa, partly driven by expanding Emergency Medicine specialist programs across the continent. Although South Africa still leads with the highest number of articles, the margins are closing with Uganda and Ethiopia closing up, as well as a healthy spread from all over. In this issue we even see a publication from war-torn Somalia describing the epidemic of conflict related injuries they see daily [1], and another addressing infectious disease outbreaks in conflict zones across Africa [2].

You will have noticed an increasing number of publications in AfJEM that talk to Out-of-Hospital-Emergency-Care (OHEC), such that we are starting a new OHEC section in the journal dedicated to this field. This is a recognition firstly that OHEC is growing in Africa as we tackle the need for acute care beyond just hospital care, driven by a combination of system maturation and policy recognition, and that there are limited options for publishing African OHEC research currently. In this issue we see the spread of OHEC covering issues as diverse as call prioritisation [3], prehospital obstetric care [4,5], critical care transfers [6], and research prioritisation in post-collision care [7].

And finally beyond OHEC, we see many submissions to our successful special issue on Education in Emergency Care which span the ever-growing Basic Emergency Care (BEC) course now expanding to Francophone Africa [8], closed-loop communications from Burundi [9], and nurses advanced life support knowledge in Ghana [10]. I’ll stop there as we plan to see this special issue pulled together in our next issue.

Enjoy your reading and we look forward to your submissions in 2026 – don’t forget to watch our webinars if you haven’t already – getting published, and becoming a reviewer available on the AfJEM website under Research here.
